# Proanthocyanidin Polymer-Rich Fraction of *Stryphnodendron adstringens* Promotes *in Vitro* and *in Vivo* Cancer Cell Death via Oxidative Stress

**DOI:** 10.3389/fphar.2018.00694

**Published:** 2018-07-03

**Authors:** Vanessa Kaplum, Anelise C. Ramos, Marcia E. L. Consolaro, Maria A. Fernandez, Tânia Ueda-Nakamura, Benedito P. Dias-Filho, Sueli de Oliveira Silva, João C. P. de Mello, Celso V. Nakamura

**Affiliations:** ^1^Programa de Pós-Graduação em Ciências Farmacêuticas, Universidade Estadual de Maringá, Maringá, Brazil; ^2^Programa de Pós-Graduação em Ciências Biológicas, Universidade Estadual de Maringá, Maringá, Brazil; ^3^Programa de Pós-Graduação em Biociências e Fisiopatologia, Universidade Estadual de Maringá, Maringá, Brazil

**Keywords:** proanthocyanidins, cervical cancer cells, oxidative stress, apoptosis, *in vivo* activity

## Abstract

Cervical cancer is the fourth most common cancer that affects women, mainly through human papilloma virus (HPV) infection with high-risk HPV16 and HPV18. The present study investigated the *in vitro* anticancer activity and mechanism of action of a proanthocyanidin polymer-rich fraction of *Stryphnodendron adstringens* (F2) in cervical cancer cell lines, including HeLa (HPV18-positive), SiHa (HPV16-positive), and C33A (HPV-negative) cells, and also evaluated *in vivo* anticancer activity. *In vitro*, cell viability was determined by the MTT assay. Cell migration was determined by the wound healing assay. The mechanism of action was investigated by performing ultrastructural analysis and evaluating reactive oxygen species (ROS) production, mitochondrial metabolism, lipoperoxidation, BCL-2 family expression, caspase expression, and DNA and cell membrane integrity. *In vivo* activity was evaluated using the murine Ehrlich solid tumor model. F2 time- and dose-dependently reduced cell viability and significantly inhibited the migration of cervical cancer cells. HeLa and SiHa cells treated with F2 (IC_50_) exhibited intense oxidative stress (i.e., increase in ROS and decrease in antioxidant species) and mitochondrial damage (i.e., mitochondrial membrane potential depolarization and a reduction of intracellular levels of adenosine triphosphate). Increases in the Bax/BCL-2 ratio and caspase 9 and caspase 3 expression, were observed, with DNA damage that was sufficient to trigger mitochondria-dependent apoptosis. Cell membrane disruption was observed in C33A cells (IC_50_ and IC_90_) and HeLa and SiHa cells (IC_90_), indicating progress to late apoptosis/necrosis. The inhibition of ROS production by *N*-acetylcysteine significantly suppressed oxidative stress in all three cell lines. *In vivo*, F2 significantly reduced tumor volume and weight of the Ehrlich solid tumor, and significantly increased lipoperoxidation, indicating that F2 also induces oxidative stress in the *in vivo* model. These findings indicate that the proanthocyanidin polymer-rich fraction of *S. adstringens* may be a potential chemotherapeutic candidate for cancer treatment.

## Introduction

Cervical cancer is the fourth most common cancer that affects women worldwide (Graham, 2017). Human papilloma virus (HPV) infection is predominantly related to cervical cancer. Nearly 70% of all cases are caused by high-risk HPV16 and HPV18 (Crosbie et al., 2013).

*Stryphnodendron adstringens* (Mart.) Coville, popularly known as “barbatimão”, is typically found in the Brazilian savannah (Albuquerque et al., 2007). Its stem bark has several biological actions, including antimicrobial activity (Ishida et al., 2006; de Freitas et al., 2018), antiprotozoan activity (Holetz et al., 2005) and antiinflammatory effects (Henriques et al., 2016). The genotoxic and acute and chronic toxicity of this plant have been assessed in rodents (Costa et al., 2010, 2013). This plant has been shown to be cytotoxic for human breast cancer cells (Sabino et al., 2017). The proanthocyanidin polymer-rich fraction of *S. adstringens* stem bark is rich in condensed tannins, or proanthocyanidins, including several flavan-3-ols, such as prodelphinidins and prorobinetinidins (de Mello et al., 1996a,b, 1999; Ishida et al., 2006).

Polyphenols, as proanthocyanidins, exhibit dual antioxidant and pro-oxidant activity, thus they are indicated for prevention and treatment of cancer, consequently (León-González et al., 2015). Pro-oxidant activity of polyphenols is generally dependent on concentration and the presence of redox-active metals, resulting in an increase of reactive oxygen species (ROS) (Pizzino et al., 2017). Low ROS levels are necessary for cell growth and proliferation, however, persistently high levels can lead to cellular oxidative injury (Moloney and Cotter, 2017). Loss of equilibrium between ROS and endogenous antioxidant species results in oxidative stress (Sosa et al., 2013). Based on its intensity, oxidative stress can induce cell death, including by apoptosis (Martindale and Holbrook, 2002; Fulda et al., 2010).

Apoptosis results from extrinsic (death receptor) or intrinsic (mitochondrial) pathways. In the extrinsic-dependent pathway, there is an interaction between death receptors and ligands, such as FasL/FasR. In the mitochondria-dependent pathway, disturbances in mitochondrial membrane integrity result from a pore-forming mechanism that is controlled by the BCL-2 family, especially pro-apoptotic Bax and anti-apoptotic BCL-2 (Sinha et al., 2013). Pro-apoptotic factors are released into the cytosol resulting in caspase 9 and caspase 3 activation, which can cause diverse cellular damage, such as DNA fragmentation, a hallmark of apoptosis (Prokhorova et al., 2015). Necrosis is characterized by the loss of cell membrane integrity, which may be attributable to intense oxidative stress and mitochondrial damage (Ryter et al., 2007).

The aim of the present study was to investigate the pro-oxidant properties of a proanthocyanidin polymer-rich fraction of *S. adstringens* (F2) through the *in vitro* anticancer activity and mechanism of action in cervical cancer cell lines, including HeLa, SiHa, and C33A cells, and also to evaluate *in vivo* anticancer activity in a murine Ehrlich solid tumor model.

## Materials and Methods

### Chemicals

The following chemicals were used: fetal bovine serum (FBS); Dulbecco’s Modified Eagle Medium (DMEM; Gibco Invitrogen); carbonylcyanide m-chlorophenylhydrazone (CCCP); 3-[4,5-dimethylthiazol-2-yl]-2,5- diphenyltetrazolium bromide (MTT); dichlorodihydrofluorescein diacetate (H_2_DCFDA); Amplex Red Hydrogen Peroxide/Peroxidase Assay Kit; 5,5-dithio-bis-(2-nitrobenzoic acid) (DTNB); tetramethylrhodamine ethyl ester (TMRE); Cell Titer-Glo Luminescent Cell Viability Assay; Hoechst 33342; diphenyl-1-pyrenylphosphine (DPPP); dimethylsulfoxide (DMSO); ethylenediaminetetraacetic acid (EDTA); *N-*acetylcysteine (NAC); sodium dodecyl sulfate (SDS) and propidium iodide (PI).

### Preparation of Proanthocyanidin Polymer-Rich Fraction

Stem bark from *S. adstringens* was collected in São Jerônimo da Serra, Paraná, Brazil, in March 2014. A voucher specimen was deposited at the herbarium of Universidade Estadual de Maringá (HUEM 28197). The bark was dried in an incubator and pulverized. A crude extract of the bark was obtained by turbo extraction in acetone:water (7:3) as described by Ishida et al. (2006). The crude extract was then filtered in a Buchner filter, and the organic solvent was removed by rotavapor and lyophilized. The proanthocyanidin polymer-rich fraction (F2) was obtained by partitioning the crude extract in water:ethyl acetate (500 ml; 1:1).

### Cell Lines and Cell Culture

The HeLa (HPV18-positive), SiHa (HPV16-positive), and C33A (HPV-negative) cervical cancer cell lines and human immortalized keratinocytes (HaCaT) were provided by Dr. Luiza L. Villa (ICESP, School of Medicine, University of São Paulo/Brazil) and Dr. Silvya S. Maria-Engler (Faculty of Pharmaceutical Sciences, University of São Paulo/Brazil). The cells were maintained at 37°C under a 5% CO_2_ atmosphere in DMEM supplemented with 10% heat-inactivated FBS and antibiotics (50 U/ml penicillin and 50 mg/ml streptomycin).

### Cell Viability Assay

Cell viability was determined by the MTT assay. HeLa, SiHa, C33A and HaCaT cells were plated at a density of 2.5 × 10^5^ cells/ml in a 96-well cell culture plate for 24 h at 37°C under a 5% CO_2_ atmosphere. The cells were treated with the proanthocyanidin polymer-rich fraction (1, 10, 25, 50, and 100 μg/ml) for 24 and 48 h at 37°C under a 5% CO_2_ atmosphere. The cells were then washed in phosphate-buffered saline (PBS), and MTT (50 μl, 2 mg/ml) was added, followed by incubation for 4 h at 37°C. Formazan crystals were solubilized in DMSO, and absorbance was read at 570 nm in a microplate reader (BioTek Power Wave XS spectrophotometer). The concentration that inhibited absorbance in 50 and 90% of the cells (IC_50_ or IC_90_, respectively) compared with the untreated control was determined by non-linear regression analysis.

### Morphology of Cervical Cancer Cell Lines

HeLa, SiHa, and C33A cells were plated at a density of 2.5 × 10^5^ cells/ml in a 24-well cell culture plate for 24 h at 37°C under a 5% CO_2_ atmosphere. The cells were pre-incubated with or without *N*-acetylcysteine (5 mM) for 2h. Afterward, the cells were treated with the proanthocyanidin polymer-rich fraction (IC_50_ and IC_90_ according to cell line) for 24 h. Cell morphology was observed under a phase-contrast inverted microscope (Olympus CKX41; 40× magnification).

### Cervical Cancer Cell Lines Wound Healing Assay

The wound healing assay evaluates cellular migration *in vitro*. HeLa, SiHa, and C33A cells were plated at a density of 2.5 × 10^5^ cells/ml in a 24-well cell culture plate for 24 h at 37°C under a 5% CO_2_ atmosphere. The cells were incubated with 0.5% heat-inactivated FBS for 6 h. The cells were scratched with a sterile 200 μl pipette tip, washed with PBS, and treated with the proanthocyanidin polymer-rich fraction (F2; IC_50_ and IC_90_ according to cell line) for 24 h at 37°C under a 5% CO_2_ atmosphere. Cell migration was observed under a phase-contrast inverted microscope (Olympus CKX41; 5× magnification).

### Transmission Electron Microscopy of Cervical Cancer

HeLa, SiHa, and C33A cells were plated at a density of 2.5 × 10^5^ cells/ml for 24 h at 37°C under a 5% CO_2_ atmosphere. The cells were pre-incubated with or without *N-*acetylcysteine (5 mM) for 2 h. The cells were treated with the proanthocyanidin polymer-rich fraction (IC_50_ and IC_90_ according to cell line) for 24 h. The cells were washed with PBS and fixed with 2.5% glutaraldehyde in 0.1 M sodium cacodylate buffer (pH 7.4) at 25°C for 2 h. The cells were postfixed with 1% osmium tetroxide and 0.8% potassium ferricyanide for 60 min at room temperature while protected from light, washed with 0.1 M sodium cacodylate buffer, and dehydrated in increasing concentrations of acetone. The material was embedded in increasing concentrations of Epon resin and polymerized at 60°C for 72 h. Thin sections were contrasted with uranyl acetate and lead citrate and observed in a JEOL JEM 1400 transmission electron microscope.

### Analysis of Cell Death by Acridine Orange and Propidium Iodide Double Staining

Cell death was analyzed by acridine orange (AO) and propidium iodide (PI) double staining. HeLa, SiHa, and C33A cells were plated at a density of 2.5 × 10^5^ cells/ml in coverslips for 24 h at 37°C under a 5% CO_2_ atmosphere. The cells were pre-incubated with or without *N-*acetylcysteine (5 mM) for 2 h. The cells were treated with the proanthocyanidin polymer-rich fraction (IC_50_ and IC_90_ according to cell line) for 24 h. The cells were washed with PBS and marked with AO (10 μg/ml) and PI (4 μg/ml) for 15 min in the dark. Fluorescent staining was analyzed using an Olympus BX51 fluorescence microscope, and images were captured using a UC30 camera within 30 min. The criteria of identification were the following: viable cells have a green nucleus and intact structure; early apoptosis appears as a bright-green nucleus with chromatin condensation; late apoptosis appears as dense orange areas of chromatin condensation; and secondary necrosis appears as a reddish-orange nucleus. The number of viable cells, early apoptotic cells, late apoptotic cells and secondary necrotic cells were determined in 200 cells, counted in triplicate.

### Reactive Oxygen Species Production in Cervical Cancer Cell Lines

The production of ROS was detected by marker H_2_DCFDA. HeLa, SiHa, and C33A cells were plated at a density of 2.5 × 10^5^ cells/ml for 24 h at 37°C under a 5% CO_2_ atmosphere. The cells were pre-incubated with or without *N-*acetylcysteine (5 mM) for 2 h. Afterward, the cells were treated with the proanthocyanidin polymer-rich fraction (IC_50_ and IC_90_ according to cell line) for 24 h. The positive control was H_2_O_2_ (200 μM). The cells were washed with PBS, marked with 10 μM H_2_DCFDA for 30 min in the dark, detached by trypsinization, and resuspended in PBS. The fluorescence intensity was quantified using a fluorescence microplate reader (VICTOR X3, PerkinElmer) at excitation and emission wavelengths of 488 and 530 nm, respectively. The fluorescence intensity was normalized to the number of cells (Souza et al., 2017).

### Hydrogen Peroxide Production in Cervical Cancer Cell Lines

The production of H_2_O_2_ was detected by Amplex RED reagent. HeLa, SiHa, and C33A cells were plated at a density of 2.5 × 10^5^ cells/ml for 24 h at 37°C under a 5% CO_2_ atmosphere. The cells were pre-incubated with or without *N-*acetylcysteine (5 mM) for 2 h. Afterward, the cells were treated with the proanthocyanidin polymer-rich fraction (IC_50_ and IC_90_ according to cell line) for 24 h. The positive control was H_2_O_2_ (200 μM). The cells were washed with PBS, detached by trypsinization, resuspended in PBS, and marked with 12 μM Amplex RED and 0.05 UI/ml horseradish peroxidase. The fluorescence intensity was quantified using a fluorescence microplate reader (VICTOR X3, PerkinElmer) at excitation, and emission wavelengths of 530 and 590 nm, respectively. The fluorescence intensity was normalized to the number of cells (Souza et al., 2017).

### Reduced Thiol Levels in Cervical Cancer Cell Lines

Reduced thiol levels were detected by DTNB reagent. HeLa, SiHa, and C33A cells were plated at a density of 2.5 × 10^5^ cells/ml for 24 h at 37°C under a 5% CO_2_ atmosphere. The cells were pre-incubated with or without *N-*acetylcysteine (5 mM) for 2 h. Afterward, the cells were treated with the proanthocyanidin polymer-rich fraction (IC_50_ and IC_90_ according to cell line) for 24 h. The positive control was H_2_O_2_ (200 μM). The cells were washed with PBS. Tris-HCl buffer (10 mM; pH 2.5) was added and sonicated. Cellular debris was removed by centrifugation. Afterward, 100 μl of the supernatant, 100 μl of phosphate buffer (500 mM; pH 7.5), and 20 μl of DTNB (1 mM) were placed in each microtiter well. Absorbance was read at 412 nm in a microplate reader (BioTek Power Wave XS spectrophotometer).

### Mitochondrial Membrane Potential and DNA Integrity Assay in Cervical Cancer Cell Lines

The mitochondrial membrane potential (ΔΨm) was detected by the marker TMRE. HeLa, SiHa, and C33A cells were plated at a density of 2.5 × 10^5^ cells/ml in 24 plates with or without coverslips for 24 h at 37°C under a 5% CO_2_ atmosphere. The cells were pre-incubated with or without *N-*acetylcysteine (5 mM) for 2 h. Afterward, the cells were treated with the proanthocyanidin polymer-rich fraction (IC_50_ and IC_90_ according to cell line) for 24 h. The positive control was CCCP (100 μM). The cells were washed with PBS, detached by trypsinization, resuspended in PBS, and marked with TMRE (25 nM) for 30 min in the dark at 37°C under a 5% CO_2_ atmosphere. The fluorescence intensity was quantified using a fluorescence microplate reader (VICTOR X3, PerkinElmer) at excitation and emission wavelengths of 540 and 595 nm, respectively. In another experiment, the cells were marked with TMRE (25 nM) and Hoechst 33342 (10 μg/ml) for 30 min in the dark. Fluorescent staining was analyzed using an Olympus BX51 fluorescence microscope, and images were captured using a UC30 camera.

### Intracellular ATP Determination in Cervical Cancer Cell Lines

Intracellular adenosine triphosphate (ATP) levels were detected by Cell Titer-Glo reagent. HeLa, SiHa, and C33A cells were plated at a density of 2.5 × 10^5^ cells/ml for 24 h at 37°C under a 5% CO_2_ atmosphere. The cells were pre-incubated with or without *N-*acetylcysteine (5 mM) for 2 h. Afterward, the cells were treated with the proanthocyanidin polymer-rich fraction (IC_50_ and IC_90_ according to cell line) for 24 h. The positive control was KCN (1000 μM). The cells were washed with PBS, detached by trypsinization, resuspended in PBS, and marked with Cell Titer-Glo reagent for 10 min at room temperature. The luminescence intensity was quantified using a fluorescence microplate reader (VICTOR X3, PerkinElmer).

### Real-Time Polymerase Chain Reaction in Cervical Cancer Cell Lines

HeLa, SiHa, and C33A cells were plated at a density of 2.5 × 10^5^ cells/ml in cell culture flasks for 24 h at 37°C under a 5% CO_2_ atmosphere. The cells were pre-incubated with or without *N-*acetylcysteine (5 mM) for 2 h. Afterward, the cells were treated with the proanthocyanidin polymer-rich fraction (IC_50_ and IC_90_ according to cell line) for 12 h. The cells were washed with PBS. RNA was extracted by the phenol-chloroform method and treated with amplification grade DNase I (Invitrogen) to remove genomic DNA contamination. Complementary DNA (cDNA) was synthesized from 1 μg of total RNA with iScript cDNA synthesis kit (Bio-Rad) using the thermal cycler (Mastercycler, Eppendorf). Real time PCR was performed with SYBR Green PCR Master Mix (Applied Byosystems) on the LightCycler 96 System (Roche Applied Science). The analysis of PCR products was performed using LightCycler 96 software (Roche Applied Science). All of the procedures were performed according to the manufacturer’s instructions. The following primer sequences were researched in the GenBank Nucleotide Database: Bax (F: 5′-TTTGCTTCAGGGTTTCATCC-3′ and R: 5′-CAGTTGAAGTTGCCGTCAGA-3′); BCL-2 (F: 5′-GCAGGCGACGAGTTTGAACT-3′ and R: 5′-GTGTCTGGTCATTTCCGACTGA-3′); Caspase 3 (F: 5′-ATACTCCACAGCACCTGGTTAT-3′ and R: 5′-AATGAGAGGGAAATACAGTACCAA-3′); Caspase 9 (F: 5′-GTACGTTGAGACCCTGGACGAC-3′ and R: 5′-GCTGCTAAGAGCCTGTCTGTCACT-3′) and 18S rRNA gene (F: 5′-GTAACCCGTTGAACCCCATT-3′ and R: 5′-CCATCCAATCGGTAGTAGCG-3′). The amplification conditions were 95°C pre-denaturation for 10 min, followed by 40 cycles at 95°C for 15 s and 60°C for 60 s. The expression levels of each sample were normalized to the housekeeping 18S rRNA gene according to the ΔCt formula (Ct_target gene_ - Ct_18SrRNA_). Relative mRNA expression levels were calculated using the 2^-ΔΔCt^ relative quantitative method, where ΔΔCt values were calculated based on ΔCt treated cells – ΔCt control cells (Schmittgen and Livak, 2008).

### DNA Fragmentation Revealed by Gel Electrophoresis in Cervical Cancer Cell Lines

HeLa, SiHa, and C33A cells were plated at a density of 2.5 × 10^5^ cells/ml for 24 h at 37°C under a 5% CO_2_ atmosphere. The cells were pre-incubated with or without *N-*acetylcysteine (5 mM) for 2 h. Afterward, the cells were treated with the proanthocyanidin polymer-rich fraction (IC_50_ and IC_90_ according to cell line) for 24 h. The cells were washed with PBS and lysed in a solution that contained Tris-HCl (10 mM: pH 8.0), EDTA (1 mM), NaCl (100 mM), SDS (0.5%), and proteinase K (20 mg/ml) at 65°C for 15 min. RNAse (1 mg/ml) was added and incubated at 37°C for 15 min. DNA was extracted by the phenol:chloroform:isoamyl alcohol method (25:24:1; v/v). DNA electrophoresis was performed on 1.0% agarose gel in Tris/boric acid buffer at 90 V for 1 h, and DNA was stained with SYBR safe DNA gel stain (Invitrogen). The molecular weight marker was the 100 bp DNA Ladder. Images were obtained using *In vivo* MS FX PRO (Carestream Molecular Imaging, Carestream Health).

### Lipid Peroxidation in Cervical Cancer Cell Lines

Lipid peroxidation was detected by the marker DPPP. HeLa, SiHa, and C33A cells were plated at a density of 2.5 × 10^5^ cells/ml for 24 h at 37°C under a 5% CO_2_ atmosphere. The cells were pre-incubated with or without *N-*acetylcysteine (5 mM) for 2 h. Afterward, the cells were treated with the proanthocyanidin polymer-rich fraction (IC_50_ and IC_90_ according to cell line) for 24 h. The positive control was H_2_O_2_ (200 μM). The cells were washed with PBS, detached by trypsinization, resuspended in PBS, and marked with DPPP (50 μm) for 15 min at room temperature. The fluorescence intensity was quantified using a fluorescence microplate reader (VICTOR X3, PerkinElmer) at excitation and emission wavelengths of 351 and 460 nm, respectively. The fluorescence intensity was normalized to the number of cells (Souza et al., 2017).

### Cell Membrane Integrity Assay in Cervical Cancer Cell Lines

Cell membrane integrity was detected by the marker PI. HeLa, SiHa, and C33A cells were plated at a density of 2.5 × 10^5^ cells/ml for 24 h at 37°C under a 5% CO_2_ atmosphere. The cells were pre-incubated with or without *N-*acetylcysteine (5 mM) for 2 h. Afterward, the cells were treated with proanthocyanidin polymer-rich fraction (IC_50_ and IC_90_ according to cell line) for 24 h. The positive control was digitonin (80 μM). The cells were washed with PBS, detached by trypsinization, resuspended in PBS, and marked with PI (4 μg/ml) for 5 min at room temperature. The fluorescence intensity was quantified using a fluorescence microplate reader (VICTOR X3, PerkinElmer) at excitation and emission wavelengths of 480 and 580 nm, respectively. The fluorescence intensity was normalized to the number of cells (Souza et al., 2017).

### *In Vivo* Antitumor Activity

Male BALB/c mice (20–25 g), 4–6 weeks of age, were obtained from Universidade Estadual de Maringá. The animals were maintained at a controlled room temperature (22°C ± 2°C) under a 12 h/12 h light/dark cycle. Food and water were available *ad libitum*. The Institutional Ethics Committee of Universidade Estadual de Maringá approved all of the procedures in this study (protocol no. 4534200417/2017).

Ehrlich ascites carcinoma (EAC) cells (1 × 10^7^ cells/ml) were injected subcutaneously in the right flanks in male BALB/c mice to obtain the Ehrlich solid tumors. After 15 days, when a palpable mass developed, the animals were randomly separated in two groups (*n* = 5/group): control group (treated with PBS vehicle) and proanthocyanidin polymer-rich fraction group (150 mg/kg/day in PBS). The treatment was administered orally by gavage and daily for 28 days. Stock solutions of F2 were prepared daily in PBS. The dimensions of Ehrlich solid tumors were measured weekly with a digital caliper (DIGIMESS^®^ 150 mm). The Ehrlich solid tumor volume (TV) was calculated by the following formula: *TV (mm^3^) = 0.52* × *A (mm)* × *[B (mm)^2^]*, where *A* and *B* are the largest and smallest diameters, respectively. On day 28 of treatment, the animals were euthanized, and the solid tumor were excised and weighed.

#### Lipid Peroxidation in Solid Tumor

Lipid peroxidation was detected by the thiobarbituric acid-reactive substances (TBARS) in terms of malondialdehyde (MDA). After euthanasia, solid tumors were weighed, sonicated, and centrifugated. The supernatant was heated in a solution that contained 0.37% thiobarbituric acid, 15% trichloroacetic acid, and 0.25 N HCl for 45 min at 95°C. Absorbance was read at 532 nm in a microplate reader (BioTek Power Wave XS spectrophotometer). Then, TBARS concentration was measured based on 𝜀 value of 153,000 M^-1^cm^-1^.

### Statistical Analysis

The data that are presented in the tables and graphs are expressed as the mean ± standard deviation of at least three independent experiments. The data were analyzed using one- or two-way analysis of variance (ANOVA) followed by Dunnett’s or Bonferroni’s *post hoc* test. Values of *p* ≤ 0.05 were considered statistically significant. The statistical analysis was performed using Prism 5 software (GraphPad, San Diego, CA, United States).

## Results

### Cytotoxic Activity in Cervical Cancer Cell Lines

**FIGURE 1:**
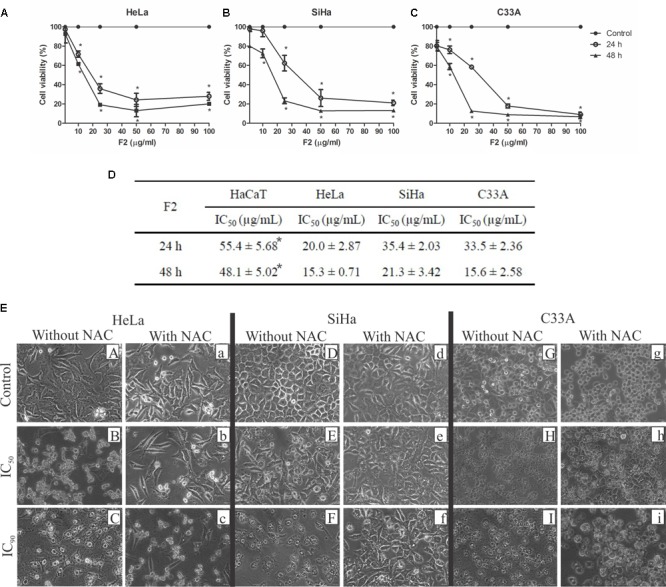
Cytotoxic activity in cervical cancer cell lines (HeLa, SiHa, and C33A cells) and human immortalized keratinocytes (HaCaT). **(A–D)** Cells were treated with the proanthocyanidin polymer-rich fraction (1–100 μg/ml) for 24 and 48 h, and cellular viability was assessed by the MTT assay. The data are expressed as the mean ± SD of three experiments that were performed in triplicate. ^∗^*p* ≤ 0.05, significant difference from control (untreated cells; two-way ANOVA followed by Bonferroni *post hoc* test). **(E)** Morphological alterations in cervical cancer cell lines (HeLa, SiHa, and C33A cells) treated with F2 (IC_50_ and IC_90_) for 24 h with or without pre-incubation with *N-*acetylcysteine (5 mM) for 2 h. ^∗^*p* ≤ 0.05, HaCaT cells significant difference from HeLa, SiHa, or C33A cell lines (one-way ANOVA followed by Dunnett’s *post hoc* test).

The proanthocyanidin polymer-rich fraction exerted time- and dose-dependent cytotoxic activity in the HeLa (HPV18-positive), SiHa (HPV16-posititve), and C33A (HPV-negative) cervical cancer cell lines (**Figures 1A–C**). The IC_50_ for HeLa, SiHa, and C33A cells was 20.0, 35.4, and 33.5 μg/ml after 24 h and 15.3, 21.3, and 15.6 μg/ml after 48 h of treatment, respectively (**Figure 1D**). After 24 h of treatment, the IC_90_ for HeLa, SiHa, and C33A cells was 64.6, 56.1, and 51.8 μg/ml, respectively. For human immortalized keratinocytes (HaCaT), the IC_50_ was 55.4 and 48.1 μg/ml at 24 and 48 h, respectively (**Figure 1D**). Thus, the cytotoxic activity of F2 was significantly higher in HeLa, SiHa, and C33A cells than in normal cells (HaCaT cells).

The morphology of HeLa, SiHa, and C33A cells treated with F2 was observed under a phase-contrast inverted microscope. All of the cell lines treated with the IC_50_ of F2 presented alterations in morphology (**Figure 1E**). Notable alterations of morphology were observed in cells treated with the IC_90_ of F2, including irregularity of shape, cellular detachment, and a bright circle around the nucleus. Cells pre-incubated with *N-*acetylcysteine (NAC) for 2 h before treatment with F2 (IC_50_ and IC_90_) presented the preservation of morphology, although an increase in cytoplasmic vacuolization was observed.

**FIGURE 2:**
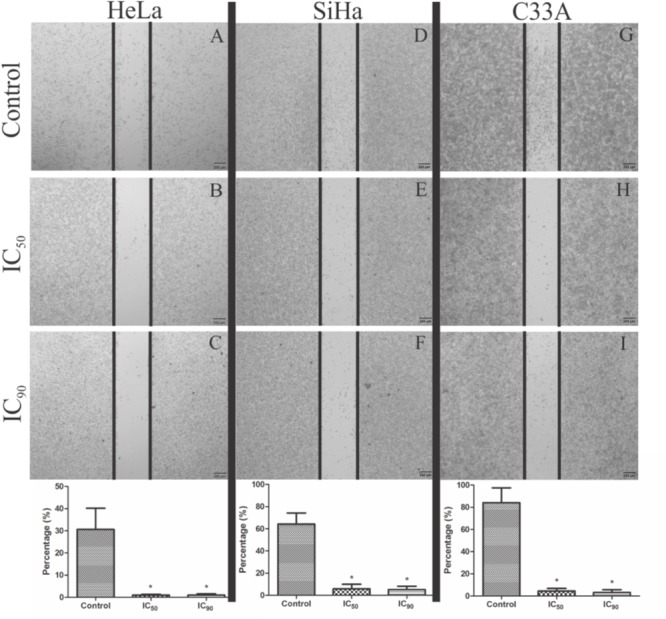
Analysis of cell migration by wound healing assay in cervical cancer cell lines (HeLa, SiHa, and C33A cells). **(A,D,G)** Control cells (untreated cells). **(B,C,E,F,H,I)** Cells treated with the proanthocyanidin polymer-rich fraction (IC_50_ or IC_90_) for 24 h and observed under a phase-contrast inverted microscope. The data are expressed as a percentage of wound closure ± SD in three experiments for HeLa, SiHa, and C33A, respectively. ^∗^*p* ≤ 0.05, significant difference from control (untreated cells; one-way ANOVA followed by Dunnett’s *post hoc* test).

Cell migration was analyzed by the wound-healing assay. HeLa, SiHa, and C33A cells treated with F2 (IC_50_ and IC_90_) presented significant inhibitions of cell migration compared with the control (**Figure 2**). For HeLa, SiHa, and C33A cells, the percentage of cell migration in control (untreated) cells was 30.6, 64.3, and 84.1%, respectively. In cells treated with F2, cell migration was less than 10% for all cell lines (**Figure 2**).

### Transmission Electron Microscopy of Cervical Cancer Cell Lines

**FIGURE 3:**
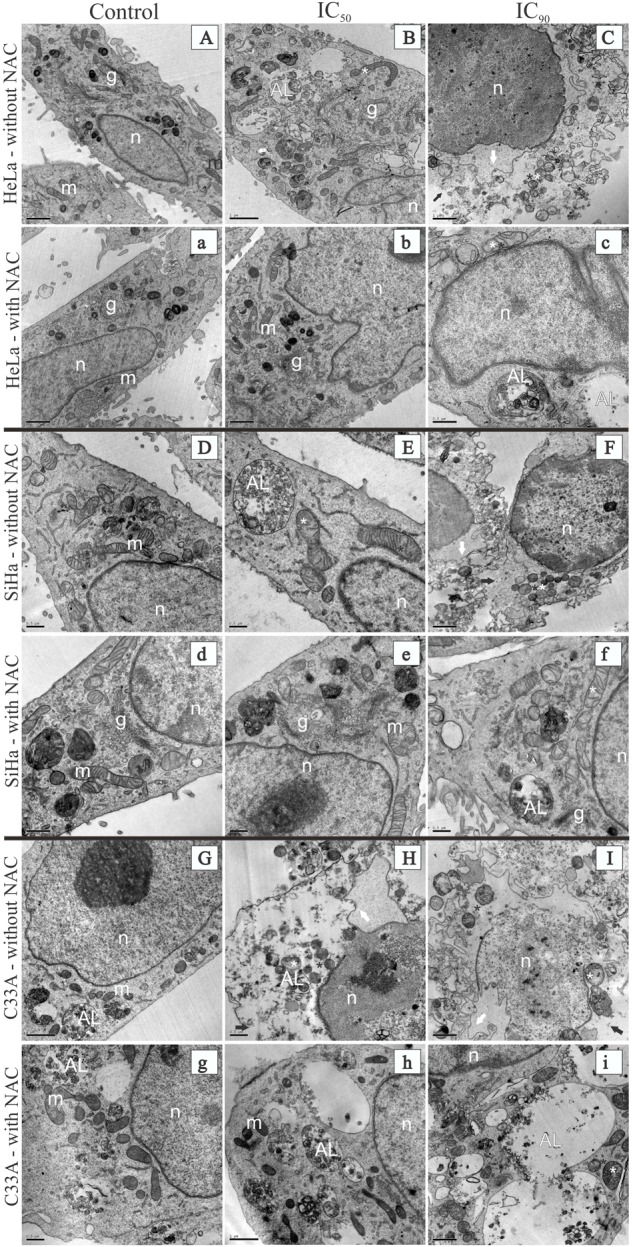
Transmission electron microscopy of cervical cancer cell lines (HeLa, SiHa, and C33A cells) treated with the proanthocyanidin polymer-rich fraction (IC_50_ or IC_90_) for 24 h. The cells were pre-incubated without **(A–I)** or with **(a–i)** *N-*acetylcysteine for 2 h before F2 treatment. HeLa, SiHa, and C33A cell control groups (untreated cells) are represented by **(A,a), (D,d)**, and **(G,g)**, respectively. HeLa, SiHa, and C33A cells treated with the IC_50_ are represented by **(B,b), (E,e)**, and **(H,h)**, respectively. HeLa, SiHa, and C33A cells treated with the IC_90_ are represented by **(C,c), (F,f)**, and **(I,i)**, respectively. Control groups (untreated cells) had a normal ultrastructure of organelles. m, mitochondria; g, Golgi apparatus; n, nucleus. HeLa. SiHa, and C33A cells that were treated with the IC_50_ or IC_90_ of F2 presented ultrastructural alterations, including mitochondrial swelling (white asterisk), a loss of mitochondrial cristae (black asterisk), cell membrane disruption (black arrow), nuclear membrane alterations (white arrow), and an autolysosome (AL). Scale bars = 0.5 μm in **(D,E,H, c–g)** and 1 μm in **(A–C,F,G,I,a,b,h,i)**.

Treatment with F2 (IC_50_) resulted in ultrastructural alterations in HeLa cells, mitochondrial swelling, and an increase in autolysosomes (**Figure 3B**). Similar alterations were observed in SiHa cells, with the additional loss of mitochondrial cristae (**Figure 3E**). HeLa and SiHa cells treated with the IC_90_ of F2 exhibited cell membrane disruption and nuclear membrane alterations (**Figures 3C,F**, respectively). C33A cells treated with the IC_50_ or IC_90_ of F2 exhibited mitochondrial swelling, a loss of mitochondrial cristae, cell membrane disruption, and nuclear membrane alterations (**Figures 3H,I**, respectively). Cells pre-incubated with NAC for 2 h before treatment with the IC_50_ of F2 exhibited preservation of the ultrastructure of mitochondria, the cell membrane, the nuclear membrane, and autolysosomes (**Figures 3b,e,h**). Treatment with the IC_90_ of F2 after pre-incubation with NAC resulted in mitochondrial swelling, a loss of mitochondrial cristae, and an increase in the number and size of autolysosomes (**Figures 3c,f,i**). The control groups of HeLa, SiHa, and C33A cells presented no ultrastructural alterations (**Figures 3A,a,D,d,G,g**).

### Analysis of Cell Death by Acridine Orange and Propidium Iodide Double Staining

The quantification of apoptotic and necrotic cells was performed by acridine orange and propidium iodide double staining, in which viable cells have a green nucleus with an intact structure, early apoptosis cells appears as a bright-green nucleus with chromatin condensation, late apoptosis appears as dense orange areas with chromatin condensation, and secondary necrosis appears as a reddish-orange nucleus (Ng et al., 2013; Hajrezaie et al., 2015).

**FIGURE 4:**
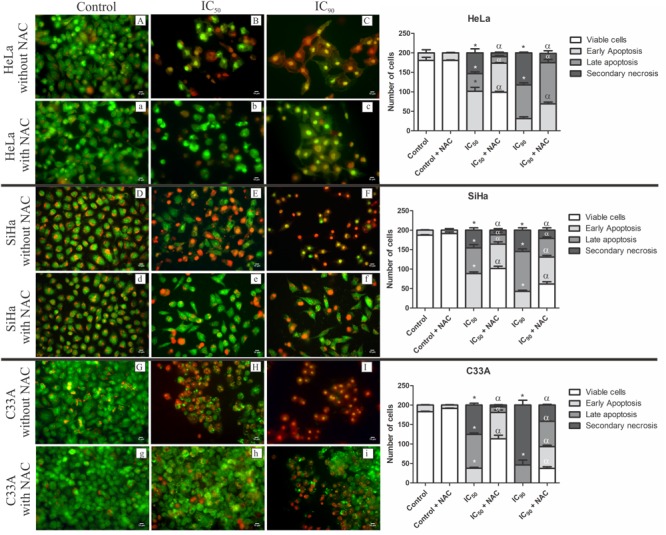
Fluorescence microscopy of cell death by acridine orange and propidium iodide double staining in cervical cancer cell lines (HeLa, SiHa, and C33A cells) treated with the proanthocyanidin polymer-rich fraction (IC_50_ or IC_90_) for 24 h. The cells were pre-incubated without **(A–I)** or with **(a–i)** *N*-acetylcysteine (5 mM) for 2h before F2 treatment. The bars represent the qualitative analysis of fluorescence micrographs determined in 200 cells in triplicate. The following criteria were applied: viable cells (green nucleus with intact structure), early apoptosis (bright-green nucleus with chromatin condensation), late apoptosis (dense orange areas with chromatin condensation), and secondary necrosis (reddish-orange nucleus). ^∗^*p* ≤ 0.05, significant difference from control (untreated cells; one-way ANOVA followed by Dunnett’s *post hoc* test); ^α^*p* ≤ 0.05, significant difference from control + NAC (one-way ANOVA followed by Dunnett’s *post hoc* test).

All of the cell lines treated with F2 (IC_50_ or IC_90_) exhibited a significant reduction of viable cells (**Figure 4**). After treatment with the IC_50_ of F2, HeLa and SiHa cells exhibited an increase in the percentage of early apoptosis (50.6 and 44.2%, respectively), late apoptosis (23 and 33%, respectively), and secondary necrosis (27 and 22.8%, respectively). Pre-incubation with NAC maintained approximately 50% of viable cells for both cell lines. After treatment with the IC_90_, an increase in the percentage of late apoptosis (43 and 51.3%, respectively) and secondary necrosis (42 and 27.3%, respectively) was observed in HeLa and SiHa cells. HeLa and SiHa cells pre-incubated with NAC and treated with the IC_90_ had 12 and 10% secondary necrosis, respectively (**Figure 4**).

After treatment with the IC_50_ or IC_90_ of F2, C33A cells exhibited an increase in the percentage of late apoptosis (43.5 and 23%, respectively) and secondary necrosis (37.6 and 77%, respectively). Cells pre-incubated with NAC and treated with the IC_50_ and IC_90_ had 56.6 and 18.5% viable cells and 2.3 and 21% secondary necrosis, respectively (**Figure 4**).

### Reactive Oxygen Species Production and Reduced Thiols Levels in Cervical Cancer Cell Lines

**FIGURE 5:**
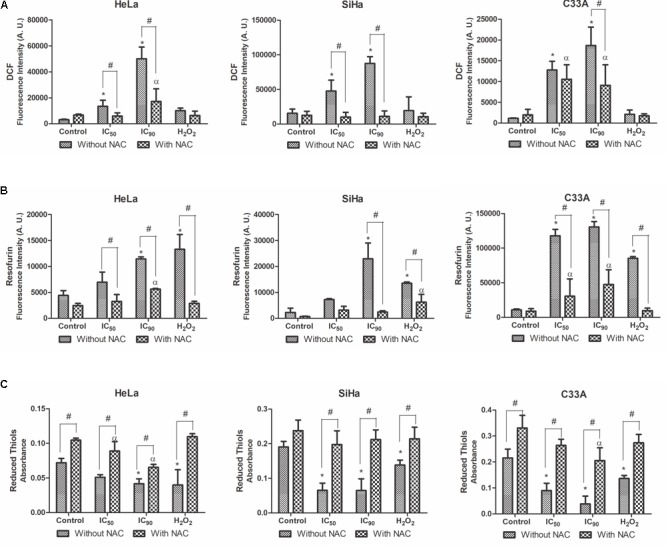
Production of reactive oxygen species (ROS) **(A)**, production of hydrogen peroxide **(B)**, and reduced thiol levels **(C)** in cervical cancer cell lines (HeLa, SiHa, and C33A cells) treated with the proanthocyanidin polymer-rich fraction (IC_50_ or IC_90_) for 24 h. The cells were pre-incubated with or without *N-*acetylcysteine (5 mM) for 2 h before F2 treatment. The positive control was H_2_O_2_ (200 μM). The data are expressed as the mean ± SD of three experiments that were performed in triplicate. ^∗^*p* ≤ 0.05, significant difference from control (untreated cells; one-way ANOVA followed by Dunnett’s *post hoc* test); ^α^*p* ≤ 0.05, significant difference from control + NAC (one-way ANOVA followed by Dunnett’s *post hoc* test); ^#^*p* ≤ 0.05, significant difference between treatment with and without NAC (two-way ANOVA followed by Bonferroni *post hoc* test).

The production of ROS in cervical cancer cell lines was analyzed by the marker H_2_DCFDA, which is cleaved by esterases and oxidized by ROS into highly green-fluorescent 2′,7′-dichlorofluorescein (DCF) (Yazdani, 2015). HeLa and SiHa cells exhibited a significant increase in ROS production after treatment with the IC_50_ (434 and 307%, respectively) and IC_90_ (1,628 and 564%, respectively; **Figure 5A**). Cells pre-incubated with the antioxidant NAC exhibited a decrease in the production of ROS after treatment with the IC_50_ and IC_90_. Similarly, C33A cells exhibited an increase in ROS production when treated the IC_90_ of F2 (1,623%), and pre-incubation with NAC prevented ROS production. The positive control, H_2_O_2_, increased ROS production.

The production of a specific type of ROS, hydrogen peroxide (H_2_O_2_), was analyzed by the marker Amplex Red; which is catalyzed by horseradish peroxidase (HRP) to produce the highly fluorescent resofurin (Debski et al., 2016). All of the cervical cancer cell lines treated with F2 exhibited an increase in H_2_O_2_ levels (**Figure 5B**). This effect was observed at a lower intensity in cells pre-incubated with NAC.

Reduced thiol levels were determined using DTNB (Ellman’s reagent). This compound uses the GSH-recycling system, where GSSG is progressively reduced to GSH by glutathione reductase and produces a color product 5-thio-2-nitrozenxoic acid, with maximal absorbance at 412 nm (Chen et al., 2008; Winther and Thorpe, 2014). A significant decrease in reduced thiol levels was observed after treating HeLa, SiHa, and C33A cells with the IC_50_ (29, 65, and 58%, respectively) and IC_90_ (42, 66, and 82%, respectively; **Figure 5C**). Less of a decrease in reduced thiol levels was observed in cells pre-incubated with NAC, especially in SiHa and C33A cells treated with the IC_50_ (17 and 20%, respectively) and IC_90_ (11 and 38%, respectively).

### Mitochondrial Membrane Potential and Intracellular ATP Levels in Cervical Cancer Cell Lines

**FIGURE 6:**
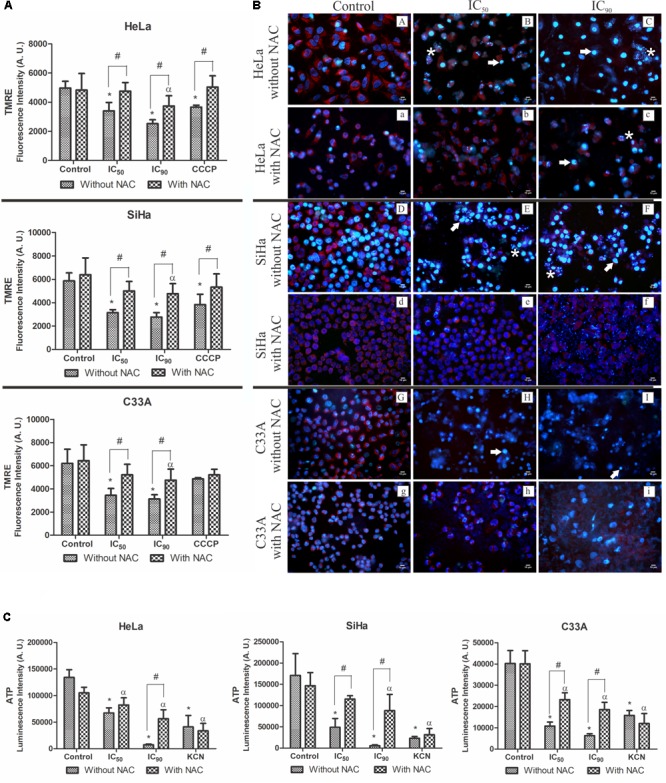
**(A)** Mitochondrial membrane potential (ΔΨm) quantification by the marker TMRE. **(B)** Fluorescence microscopy analysis of ΔΨm by the marker TMRE (red fluorescence) and DNA integrity analysis by the marker Hoechst 33342 (blue fluorescence), showing DNA fragmentation (white asterisk) and chromatin condensation (white arrow). **(C)** Intracellular ATP levels were determined by Cell Titer-GLO reagent. Cervical cancer cell lines (HeLa, SiHa, and C33A cells) were treated with the proanthocyanidin polymer-rich fraction (IC_50_ or IC_90_) for 24 h. The cells were pre-incubated with or without *N-*acetylcysteine (5 mM) for 2 h before F2 treatment. The positive control was CCCP (100 μM) and KCN (1000 μM). The data are expressed as the mean ± SD of three experiments that were performed in triplicate. ^∗^*p* ≤ 0.05, significant difference from control (untreated cells; one-way ANOVA followed by Dunnett’s *post hoc* test); ^α^*p* ≤ 0.05, significant difference from control + NAC (one-way ANOVA followed by Dunnett’s *post hoc* test); ^#^*p* ≤ 0.05, significant difference between treatment with and without NAC (two-way ANOVA followed by Bonferroni *post hoc* test).

Considering the ultrastructural alterations of mitochondria in HeLa, SiHa, and C33A cells treated with F2, we analyzed ΔΨm using the cationic fluorophore TMRE. This marker accumulates in active mitochondria and emits red fluorescence in polarized mitochondria (Walsh et al., 2017). All of the cells treated with F2 (IC_50_ or IC_90_) exhibited a significant dose-dependent decrease in ΔΨm compared with the control (untreated cells). ΔΨm loss was 31.5, 46.3, and 44.4% in HeLa, SiHa, and C33A cells that were treated with the IC_50_ and 49.1, 52.7, and 49.5% in cells that were treated with the IC_90_, respectively. The ΔΨm of cells pre-incubated with NAC underwent less depolarization. The membrane uncoupler CCCP induced the depolarization of ΔΨm (**Figure 6A**).

Similar results were observed when ΔΨm was observed under a fluorescence microscope (**Figure 6B**). Control cells (untreated cells; **Figure 6B,A,a,D,d,G,g**) exhibited intense red fluorescence, typically found in polarized mitochondria. After treatment with F2 (IC_50_ or IC_90_), red fluorescence significantly decreased. Cells pre-incubated with NAC still presented red fluorescence.

**Figure 6C** shows intracellular ATP levels, determined by Cell Titer-Glo reagent, in cervical cancer cell lines. A significant decrease in intracellular ATP levels was observed in HeLa, SiHa, and C33A cells treated with the IC_50_ (49.7, 71.4, and 73.2%, respectively) and IC_90_ (94.4, 96.7, and 84.3%, respectively) of F2. In cells pre-incubated with NAC and treated with the IC_90_, intracellular ATP levels decreased by 46.2, 39.8, and 53.8% in HeLa, SiHa, and C33A cells, respectively. The positive control, KCN, decreased luminescence (**Figure 6C**).

### Bax, BCL-2, Caspase 9 and Caspase 3 Expression in Cervical Cancer Cell Lines

**FIGURE 7:**
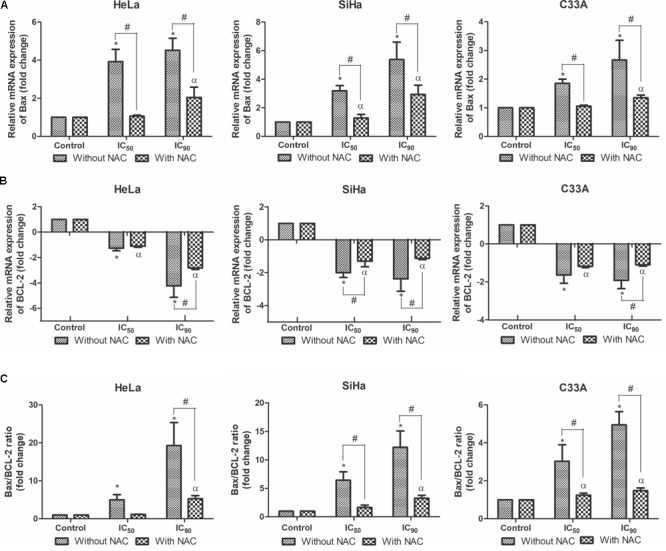
Real-time PCR of relative mRNA expression levels of Bax **(A)**, BCL-2 **(B)** and Bax/BCL-2 ratio **(C)** in cervical cancer cell lines (HeLa, SiHa, and C33A cells) treated with the proanthocyanidin polymer-rich fraction (IC_50_ or IC_90_) for 12 h. The cells were pre-incubated with or without *N-*acetylcysteine (5 mM) for 2 h before F2 treatment. The data are expressed as the mean ± SD of two experiments that were performed in duplicate. ^∗^*p* ≤ 0.05, significant difference from control (untreated cells; one-way ANOVA followed by Dunnett’s *post hoc* test); ^α^*p* ≤ 0.05, significant difference from control + NAC (one-way ANOVA followed by Dunnett’s *post hoc* test); ^#^*p* ≤ 0.05, significant difference between treatment with and without NAC (two-way ANOVA followed by Bonferroni *post hoc* test).

BCL-2 family proteins play an important role in regulating apoptosis as promoters or inhibitors of the cell death process (Hata et al., 2015). Real-time PCR was used to evaluate mRNA expression levels of pro-apoptotic Bax and anti-apoptotic BCL-2. HeLa, SiHa, and C33A cells treated with F2 (IC_50_ or IC_90_) exhibited a dose-dependent upregulation of Bax mRNA expression levels (**Figure 7A**). All of the cell lines exhibited a dose-dependent downregulation of BCL-2 mRNA expression levels (**Figure 7B**), consequently increasing the Bax/BCL-2 ratio (**Figure 7C**). Cells pre-incubated with NAC exhibited less Bax mRNA expression and more BCL-2 mRNA expression, suggesting that *N*-acetylcysteine protected the cells.

**FIGURE 8:**
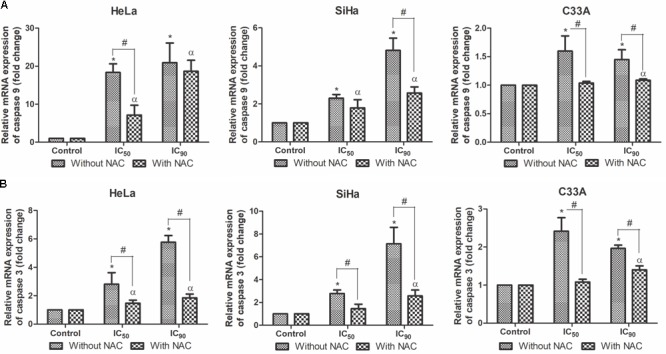
Real-time PCR of relative mRNA expression levels of caspase 9 **(A)** and caspase 3 **(B)** in cervical cancer cell lines (HeLa, SiHa, and C33A cells) treated with the proanthocyanidin polymer-rich fraction (IC_50_ or IC_90_) for 12 h. The cells were pre-incubated with or without *N-*acetylcysteine (5 mM) for 2 h before F2 treatment. The data are expressed as the mean ± SD of two experiments that were performed in duplicate. ^∗^*p* ≤ 0.05, significant difference from control (untreated cells; one-way ANOVA followed by Dunnett’s *post hoc* test); ^α^*p* ≤ 0.05, significant difference from control + NAC (one-way ANOVA followed by Dunnett’s *post hoc* test); ^#^*p* ≤ 0.05, significant difference between treatment with and without NAC (two-way ANOVA followed by Bonferroni *post hoc* test).

The central machinery of the apoptotic cascade involves caspases, such as caspase 9 and caspase 3 (Prokhorova et al., 2015). All of the cell lines treated with F2 for 12 h exhibited a dose-dependent upregulation of caspase 9 and caspase 3 mRNA expression levels, especially SiHa cells (**Figures 8A,B**). All of the cell lines pre-incubated with NAC exhibited equal or less caspase 9 and caspase 3 mRNA expression levels compared with cell treated only with F2.

### DNA Fragmentation in Cervical Cancer Cell Lines

**FIGURE 9:**
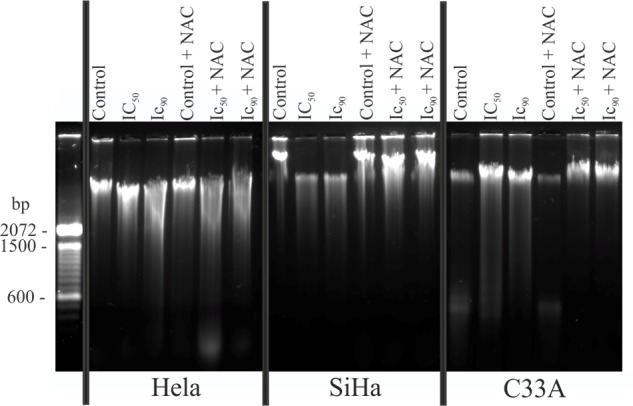
DNA fragmentation was evaluated by agarose gel electrophoresis in cervical cancer cell lines (HeLa, SiHa, and C33A cells) treated with the proanthocyanidin polymer-rich fraction (IC_50_ or IC_90_) for 24 h. The cells were pre-incubated with or without *N-*acetylcysteine (5 mM) for 2 h before F2 treatment. The experiments were performed in triplicate, yielding similar results.

Caspases promote DNA fragmentation, a hallmark of apoptosis (Larsen and Sørensen, 2017). DNA fragmentation, determined by agarose gel electrophoresis, was observed in HeLa, SiHa, and C33A cells after treatment with the IC_50_ or IC_90_ of F2 compared with control cells (**Figure 9**). Similar observations were found with fluorescence microscopy with Hoechst 33342, in which chromatin condensation was also observed (**Figure 6B**). Cells pre-incubated with NAC exhibited little DNA fragmentation (**Figure 9**).

### Lipoperoxidation and Cell Membrane Integrity in Cervical Cancer Cell Lines

**FIGURE 10:**
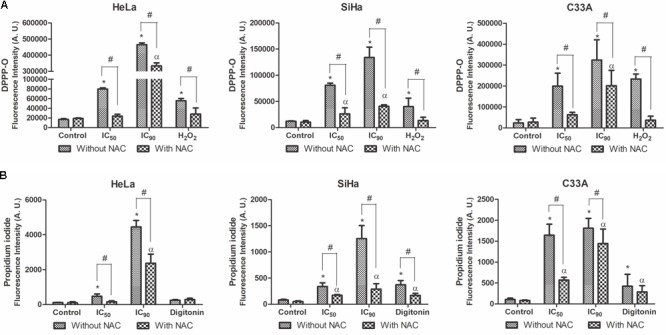
Lipoperoxidation **(A)** and cell membrane integrity **(B)** in cervical cancer cell lines (HeLa, SiHa, and C33A cells) treated with the proanthocyanidin polymer-rich fraction (IC_50_ or IC_90_) for 24 h. The cells were pre-incubated with or without *N-*acetylcysteine (5 mM) for 2 h before F2 treatment. The positive control was H_2_O_2_ (200 μM) and digitonin (80 μM). The data are expressed as the mean ± SD of three experiments performed in triplicate. ^∗^*p* ≤ 0.05, significant difference from control (untreated cells; one-way ANOVA followed by Dunnett’s *post hoc* test); ^α^*p* ≤ 0.05, significant difference from control + NAC (one-way ANOVA followed by Dunnett’s *post hoc* test); ^#^*p* ≤ 0.05, significant difference between treatment with and without NAC (two-way ANOVA followed by Bonferroni *post hoc* test).

Lipoperoxidation was analyzed by DPPP marker, which reacts with lipid hydroperoxides to generate fluorescent DPPP oxide (Okimoto et al., 2000). F2 treatment significantly increased lipid peroxidation in HeLa, SiHa, and C33A cells treated with the IC_50_ (472, 670, and 840%, respectively) and IC_90_ (2,760, 1,110, and 1,360%, respectively). Pre-incubation with NAC resulted in a small increase in lipoperoxidation in cells treated with F2 (**Figure 10A**). The positive control, H_2_O_2_, increased lipoperoxidation.

Cell membrane integrity was analyzed by the marker PI. A significant loss of cell membrane integrity was observed in HeLa, SiHa, and C33A cells treated with the IC_50_ (413, 418, and 1,540%, respectively) and IC_90_ (3,880, 1,550, and 1,700%, respectively) of F2 (**Figure 10B**). Cells pre-incubated with NAC exhibited less damage to the cell membrane, especially when treated with the IC_50_. The positive control, digitonin, induced damage to the cell membrane.

### *In Vivo* Antitumor Activity

**FIGURE 11:**
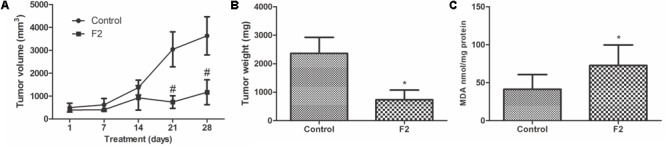
*In vivo* anticancer activity of proanthocyanidin polymer-rich fraction (F2) in BALB/c mice with Ehrlich solid tumors. Treatment included control group (untreated control) and F2 group (150 mg/kg/day). Both treatments were administered orally and daily for 28 days. **(A)** The dimensions of Ehrlich solid tumors were recorded weekly by calculating the tumor volume. **(B)** Ehrlich solid tumors were weighted after the animal were euthanized. **(C)** Lipid peroxidation by TBARS in terms of MDA. The data are expressed as the mean ± SD (*n* = 5/group). ^∗^*p* ≤ 0.05, significant difference between control and F2 (Mann–Whitney test); ^#^*p* ≤ 0.05, significant difference between control and F2 (two-way ANOVA followed by Bonferroni *post hoc* test).

*In vivo* antitumor activity of the proanthocyanidin polymer-rich fraction (F2) was observed in the murine Ehrlich solid tumor model, in which BALB/c mice were treated with 150 mg/kg/day F2 for 28 days. The dose was selected according to previous acute and chronic toxicity studies in rodents (Costa et al., 2010, 2013). No animals died in neither the control group nor in the F2 group (data not shown). The tumor volume significantly decreased after treatment, with 67.8% tumor growth inhibition (**Figure 11A**). F2 significantly reduced tumor weight compared with the control, with a 68.8% reduction of tumor weight (**Figure 11B**).

Lipid peroxidation was performed in the tumor to investigate the influence of oxidative stress, through detection of TBARS in terms of MDA (Pompella et al., 1987; Kudryavtseva et al., 2016). After treatment with F2, there was a significant increase in MDA, thus treatment induced lipoperoxidation in the tumor (**Figure 11C**).

## Discussion

The proanthocyanidin polymer-rich fraction of *S. adstringens* (F2) time- and dose-dependently reduced the viability of HeLa (HPV18-positive), SiHa (HPV16-positive), and C33A (HPV-negative) cell lines. *In vitro*, F2 treatment (IC_50_) induced intense oxidative stress and mitochondrial damage that were sufficient to trigger mitochondria-dependent apoptosis. Treatment with the high concentration of F2 (IC_90_) resulted in progression to late apoptosis/necrosis. The synthetic antioxidant *N-*acetylcysteine significantly suppressed oxidative stress. In the murine Ehrlich solid tumor model, F2 exerted potent *in vivo* anticancer activity.

Polyphenols, such as proanthocyanidins, are widely investigated as cancer therapies (Nandakumar et al., 2008; Zhou et al., 2016). Here, proanthocyanidin polymer-rich fraction of *S. adstringens* (F2) treatment time- and dose-dependently reduced the viability of HeLa, SiHa, and C33A cells. Grape seed proanthocyanidins treatment showed similar cytotoxic activity in cervical cancer cells (Chen et al., 2014). It should be highlighted that F2 was significantly less toxic for HaCaT cells than cervical cancer cells. Furthermore, the F2 cytotoxic concentration for 50% cells (CC_50_) was the same 125, 150, and >1,000 μg/ml for murine macrophages (J774G8 cells), green monkey kidney (Vero cells) and red blood cells, respectively (Ishida et al., 2006).

Cancer cell migration and invasion are the first steps of metastasis (Clark and Vignjevic, 2015). In the wound healing assay with HeLa, SiHa, and C33A cells, F2 treatment significantly inhibited cancer cell migration. The morphology of all three cell lines showed intense alterations, such as the irregularity of shape, cellular detachment, and a bright circle around the nucleus.

Morphological analysis by transmission electron microscopy is considered the “gold standard” for understanding and classifying cell death (Vanden Berghe et al., 2013). Generally, in apoptosis, cells undergo fragmentation and condensation of the nucleus and the swelling of organelles, such as mitochondria. Apoptotic cells may undergo a late process of secondary necrosis, characterized by cell membrane disruption (Wong, 2011). In addition to TEM, OA/PI double staining was performed to classify cell death. HeLa and SiHa cells treated with F2 (IC_50_) exhibited intense nuclear and mitochondrial changes and increase in the percentage of early apoptotic cells in the AO/PI assay. HeLa and SiHa cells treated with the IC_90_ also resulted in cell membrane disruption, which is characteristic of late apoptosis and secondary necrosis that were also observed in the AO/PI assay. In C33A cells, all of the aforementioned changes in TEM and AO/PI double staining were observed at both concentrations of F2, indicating late apoptosis and secondary necrosis.

However, the synthetic antioxidant *N-*acetylcysteine preserved organelle ultrastructure and increased cell viability in TEM and in OA/PI double staining, respectively. NAC scavenges ROS and increases GSH synthesis, likely increasing viable cells (Zafarullah et al., 2003). For this reason, we evaluated the oxidative stress in all three cell lines.

Oxidative stress is defined as the lack of equilibrium between ROS and endogenous antioxidant species (Sosa et al., 2013). ROS are reactive and short-lived molecules that contains unpaired electrons, as hydroxyl (OH^∙^) and non-radical ROS, as hydrogen peroxide (H_2_O_2_) (Moloney and Cotter, 2017). Endogenous antioxidants include the thiol-based intracellular antioxidant glutathione (GSH), the most abundant ROS scavenging agent in the cell (Dickinson and Forman, 2002; Sosa et al., 2013). Cancer cells are distinguished by high levels of oxidative stress. Thus, ROS accumulation in cancer cells is more harmful than in normal cells. Therefore, an increase in oxidative stress induces death preferentially in cancer cells (Gorrini et al., 2013; Panieri and Santoro, 2016).

HeLa, SiHa, and C33A cells treated with F2 exhibited a significant dose-dependent increase in ROS generation and a significant decrease in reduced thiols levels, indicating that intense oxidative stress triggered cell death. This suggests that the pro-oxidant properties of F2 promotes cell death mechanisms in oxidative stress-sensitive cancers, such as cervical cancer. Similarly, other polyphenols can also induce oxidative stress (Mileo and Miccadei, 2016; Mao et al., 2017). Furthermore, preincubation with antioxidant NAC reverted oxidative stress, confirming that this effect mediated cancer cell death. NAC preincubation also prevents oxidative stress in other cancer cells (Chang et al., 2017; Shiau et al., 2017).

Mitochondria are major sources of intracellular ROS, and a disproportional increase in ROS production, as in oxidative stress, can result in mitochondrial dysfunction (Redza-Dutordoir and Averill-Bates, 2016). Disturbances in membrane integrity can result in mitochondria-mediated apoptosis, usually manifested by ΔΨm depolarization and a decrease in ATP levels (Sinha et al., 2013). In the present study, all three cell lines treated with F2 exhibited significant dose-dependent decrease in ΔΨm and intracellular ATP levels. However, lower depolarization of ΔΨm and higher levels of intracellular ATP were observed in cells pre-incubated with NAC. This indicates that the synthetic antioxidant protected mitochondrion integrity from ROS-induced damage in cells treated with the IC_50_ of F2. Reactive oxygen species production was incompletely scavenged by NAC when the cells treated with the IC_90_, revealed by TEM micrographs. *S. adstringens* induced mitochondrial damage in isolated rat liver mitochondria, apparently due to uncoupling of oxidative phosphorylation, inhibition of mitochondrial electron transport and inhibition of ATP-synthase (Rebecca et al., 2003).

Considering the mitochondrial depolarization, we evaluated the mitochondrial membrane integrity. Mitochondrial depolarization results from a pore-forming mechanism controlled by the BCL-2 family, especially the pro-apoptotic Bax and anti-apoptotic BCL-2 (Sinha et al., 2013). An increase in Bax protein levels leads to the formation of multimeric pores in the mitochondrial membrane, resulting in the release of pro-apoptotic factors into the cytosol. BCL-2 protein prevents this release, thus protecting the cell from apoptosis (Hata et al., 2015). The Bax/BCL-2 ratio is critical for the induction of apoptosis, and a significant increase in the Bax/BCL-2 ratio was observed after F2 treatment. Cells pre-incubated with NAC resulted in a decrease in the Bax/BCL-2 ratio, reducing the susceptibility of cells to apoptosis.

In the treatment with F2, mitochondrial damage associated with the increase of Bax/BCL-2 ratio likely induced mitochondrial-dependent pathway of apoptosis. In this pathway, pro-apoptotic factors are released into the cytosol, including cytochrome c, which binds to apoptotic protease activating factor (Apaf-1) and pro-caspase 9 to form the apoptosome that activates caspase 3, resulting in cellular damage, such as DNA fragmentation (Ashkenazi et al., 2017). HeLa, SiHa, and C33A cells treated with F2 exhibited a dose-dependent upregulation of caspase 9 and caspase 3 mRNA expression levels. All three cell lines pre-incubated with NAC exhibited equal or less caspase 9 and caspase 3 mRNA expression levels compared with cells treated only with F2, thus confirming the protective effect of *N*-acetylcysteine due to oxidative stress intensity reduction.

The increase of caspase 3 results in DNA fragmentation, which is a hallmark of apoptosis (Prokhorova et al., 2015). All three cell lines underwent DNA fragmentation, revealed by agarose gel electrophoresis and the marker Hoechst 33342, after treatment with F2, confirming an apoptotic process. DNA fragmentation also was observed in human breast cancer cells treated with *S. adstringens* leaf fraction (Sabino et al., 2017).

Furthermore, oxidative stress induces lipoperoxidation, specifically through lipid hydroperoxides that affect lipid-containing structures (Kudryavtseva et al., 2016). F2 treatment significantly increased lipoperoxidation in all three cell lines. The excess accumulation of lipid hydroperoxides under depletion of reduced thiol levels and intracellular ATP can promote cell membrane disruption, resulting in cells with typical necrosis morphology (Higuchi, 2004; Ryter et al., 2007; Rasola and Bernardi, 2011). Thus, an intense cell membrane disruption was observed after treatment with the IC_50_ of F2 in C33A cells and treatment with the IC_90_ in all three cell lines, as revealed by transmission electron microscopy and OA/PI double staining. Lipoperoxidation was lower in cells preincubated with NAC, as well as the damage in cell membrane. These results suggest that the ROS scavenger protected all the cell lines by reducing oxidative stress intensity.

HeLa and SiHa cells contain integrated HPV18 and HPV16 DNA, respectively (Ding et al., 2007). In these cells, F2 treatment induced intense oxidative stress and mitochondrial damage, and cell membrane disruption was observed at the high concentration (IC_90_). C33A cells are HPV-negative and contain mutant p53 (Ding et al., 2007). In C33A cells, in addition to oxidative stress, notable cell membrane disruption was found with both treatments. The different cellular responses are likely attributable to different basal levels of ROS and antioxidant species in each cell line (Filippova et al., 2014).

*In vivo* tumor models are essential for the evaluation of possible chemotherapies (Jung, 2014). Ehrlich solid tumors provide an efficient way to investigate chemotherapeutic agents (Noaman et al., 2008; Biswas et al., 2016; Srivastava et al., 2016). This tumor is formed through the subcutaneous inoculation of Ehrlich ascites carcinoma cells, a breast adenocarcinoma (Ozaslan et al., 2013). Based on promising *in vitro* results, F2 was evaluated in a murine Ehrlich solid tumor model. The administration of 150 mg/kg/day F2 in mice significantly reduced tumor volume and tumor weight. Synergism between vanillin and doxorubicin showed similar results in Ehrlich solid tumors (Elsherbiny et al., 2016). *In vitro*, F2 induced oxidative stress in cancer cells, resulting in intense lipoperoxidation. Similarly, there was also lipoperoxidation increase in tumors treated with F2, indicating that F2 also induces oxidative stress in the *in vivo* model.

Previous work has shown that polyphenols may trigger DNA damage and lipoperoxidation due to ROS generation (León-González et al., 2015). Furthermore, suppression of endogenous antioxidants leads to cancer cells sensitization to chemo- and radiotherapy (Sznarkowska et al., 2017). Considering that pro-oxidant activity of F2 induced oxidative stress, proanthocyanidins could potentially lead to a new group of natural chemotherapeutic agents, either used alone or in combination therapy to reduce drug resistance. Thus, natural plant-based polyphenolic compounds are promising as future cancer chemotherapeutic agents (León-González et al., 2015; Redondo-Blanco et al., 2017).

## Conclusion

The proanthocyanidin polymer-rich fraction of *S. adstringens* time- and dose-dependently reduced cell viability and the migration of the cervical cancer cell lines HeLa (HPV-18 positive), SiHa (HPV-16 positive), and C33A (HPV-negative). In HeLa and SiHa cells, F2 treatment (IC_50_) induced intense oxidative stress and mitochondrial damage sufficient to trigger mitochondria-dependent apoptosis. Moreover, cell membrane disruption was observed in C33A cells (IC_50_ and IC_90_) and HeLa and SiHa cells (IC_90_), indicating progression to late apoptosis/necrosis. The inhibition of ROS production by the synthetic antioxidant NAC significantly suppressed oxidative stress in all three cell lines. Anticancer activity *in vivo* of F2 in the Ehrlich solid tumor showed significantly reduced tumor volume and weight. Similar to the *in vitro* results, F2 significantly increased lipoperoxidation, indicating that F2 also induces oxidative stress in the *in vivo* model. In conclusion, the proanthocyanidin polymer-rich fraction of *S. adstringens* may be a potential chemotherapeutic candidate for cancer treatment.

## Author Contributions

VK conception, design and execution of the experimental studies, analysis and interpretation of the results, draft and writing of the manuscript. AR conception and execution of the experimental studies. MC, MF, TU-N, BD-F, SS, and JdM conception and design of the study, critical review and correction of the manuscript. CN coordination of all stages of the work, conception, design and draft the study, critical review and correction of the manuscript.

## Conflict of Interest Statement

The authors declare that the research was conducted in the absence of any commercial or financial relationships that could be construed as a potential conflict of interest.
